# Handgrip strength across clinical conditions and health-related outcomes in older adults: a scoping review

**DOI:** 10.3389/fragi.2026.1804033

**Published:** 2026-06-30

**Authors:** José Julián Bernal-Sánchez, Duber Esteban Uzuriaga-Fori, Sharon Estefany Rivera-Viveros, Zharick Marcela Viafara-Carabali, Esther Cecilia Wilches-Luna, Diana Yazmín Perafan-Gonzalez

**Affiliations:** 1 Facultad de Salud, Programa de Fisioterapia, Universidad Santiago de Cali, Cali, Colombia; 2 Grupo de Investigación Salud y Movimiento, Universidad Santiago de Cali, Cali, Colombia; 3 Facultad de Salud, Escuela de Rehabilitación Humana, Universidad del Valle, Cali, Colombia; 4 Grupo de Investigación Ejercicio y Salud Cardiopulmonar (GIESC), Universidad del Valle, Cali, Colombia

**Keywords:** aged, cognition, frailty, hand strength, health status, muscle strength

## Abstract

**Background and Aim:**

Handgrip strength (HGS) is a simple, non-invasive, and widely used measure of muscle function that has been examined in relation to several health-related outcomes in older adults. This scoping review aimed to map the extent, range, and nature of the evidence on the relationship between HGS and clinical conditions or health-related outcomes in older adults and aging-related populations, with particular attention to population characteristics, outcome classification, and HGS measurement protocols.

**Methods:**

This scoping review was reported in accordance with the PRISMA-ScR checklist. Observational studies, including cross-sectional, cohort, longitudinal, and panel designs, were eligible when they examined HGS in relation to clinical conditions or health-related outcomes in older adults or aging-related populations. Searches were conducted in ScienceDirect, Scopus, Web of Science, LILACS, SciELO, and MEDLINE via PubMed, with SpringerLink used as a supplementary publisher platform. Articles published in English, Spanish, or Portuguese were considered. Study selection, data charting, and methodological appraisal were performed using structured forms. Findings were synthesized descriptively by study characteristics, health-related domain, HGS measurement protocol, and HGS operationalization.

**Results:**

The search identified 890 records, and 18 studies comprising 243,334 participants were included in the final synthesis. Ten studies were cross-sectional, whereas eight used longitudinal, cohort, or panel designs. Neurocognitive and mental health outcomes were the most frequently examined domain, followed by musculoskeletal conditions or syndromes, functional outcomes, quality of life, frailty, comorbidity burden, and cardiovascular risk. HGS measurement varied across studies in terms of dynamometer type, hand assessed, number of attempts, summary value, cut-off points, and analytical operationalization. Lower HGS or HGS-related parameters were reported across studies in relation to poorer health-related outcomes, although study design, population heterogeneity, and differences in HGS protocols limited direct comparability.

**Conclusion:**

This review shows that HGS has been studied across multiple aging-related health domains, particularly neurocognitive and musculoskeletal outcomes. However, the predominance of observational designs and heterogeneity in HGS measurement and outcome definitions limit causal interpretation. Further longitudinal studies using standardized HGS protocols and consistent outcome definitions are needed.

## Introduction

Population aging is one of the leading global public health challenges (Rodrigues et al., 2022). As life expectancy continues to rise, older adults are increasingly affected by chronic diseases, geriatric syndromes, cognitive decline, musculoskeletal conditions, and functional limitations. These health-related conditions and outcomes may compromise independence and functional capacity and are associated with hospitalization, institutionalization, mortality, reduced quality of life, and increased burden on health systems (Greco et al., 2019).

In this context, the identification and standardized interpretation of simple functional measures have become increasingly relevant in clinical, research, and public health settings. Handgrip strength (HGS) has emerged as a simple, non-invasive, feasible, and widely used measure of muscle function, with increasing relevance for health screening, surveillance, and the interpretation of functional status across adulthood (Tomkinson et al., 2025). Numerous studies have reported associations between lower HGS and disability, functional decline, geriatric syndromes, and mortality, supporting its potential value as part of comprehensive geriatric assessment (Concha-Cisternas et al., 2021).

However, the literature linking HGS with health status in older adults remains dispersed across different clinical conditions, geriatric syndromes, functional outcomes, and adverse health events. Previous systematic reviews and meta-analyses have generally focused on specific outcomes, such as cognitive impairment, depression, mobility limitations, type 2 diabetes, metabolic syndrome, or all-cause mortality. Although these reviews support the clinical relevance of HGS, they also show substantial heterogeneity in study design, population characteristics, outcome definitions, and HGS measurement protocols (Rijk et al., 2016; Zasadzka et al., 2021).

An evidence-mapping approach is therefore needed to clarify the extent, range, and nature of the available literature, to distinguish the types of clinical conditions and health-related outcomes that have been studied, and to identify methodological gaps in HGS measurement and reporting that may limit comparability across studies and settings. This may be particularly relevant for underrepresented regions, including Latin America (Ramírez-Vélez et al., 2019), where population-specific evidence on HGS and aging-related health outcomes may be less consistently represented in international syntheses (Kunutsor et al., 2021; Lee, 2020; Rijk et al., 2016; Zasadzka et al., 2021).

Based on these considerations, this scoping review aimed to map the extent, range, and nature of the evidence on the relationship between HGS and clinical conditions or health-related outcomes in older adults, and to identify methodological gaps related to population characteristics, outcome classification, and HGS measurement protocols.

## Methods

### Study design

This scoping review was designed as an evidence-mapping review to describe the extent, range, and nature of published studies examining HGS in relation to clinical conditions or health-related outcomes in older adults and aging-related populations. The review is reported in accordance with the PRISMA-ScR checklist. The review protocol was not registered in any publicly accessible registry.

The review question was: What is the extent, range, and nature of the evidence on the relationship between HGS and clinical conditions or health-related outcomes in older adults, and what methodological gaps exist regarding population characteristics, outcome classification, and HGS measurement protocols?

### Eligibility criteria

Eligibility criteria were defined using the PCC framework (Population, Concept, Context). The population of interest was older adults. Studies including mixed-age samples were also considered eligible when they explicitly included older adults or aging-related populations and addressed clinical conditions or health-related outcomes relevant to aging. Studies focused predominantly on general adult or middle-aged clinical populations without an aging-related focus or without extractable evidence relevant to older adults were excluded from the main synthesis.

The concept was HGS assessed using dynamometry or another objective grip-strength measurement method. The context was the study of HGS in relation to clinical conditions or health-related outcomes, including neurocognitive, mental health, musculoskeletal, cardiometabolic, functional, quality-of-life, frailty-related, comorbidity-burden, or adverse health outcomes.

Observational studies, including cross-sectional, cohort, longitudinal, and panel designs, were eligible for inclusion. Clinical trials were considered eligible only if they reported baseline or observational associations between HGS and a clinical condition or health-related outcome relevant to the review question. Articles published in English, Spanish, or Portuguese were included, with no restrictions on publication year. Review articles, qualitative studies, conference abstracts, theses, unpublished reports, and grey literature were excluded.

### Sources of information and search strategy

The search was conducted in ScienceDirect, Scopus, Web of Science, LILACS, SciELO, and MEDLINE via PubMed. SpringerLink was used as a supplementary publisher platform to broaden retrieval of potentially relevant full-text articles from Springer journals and was not treated as a bibliographic database. Searches were conducted in March 2024. The search strategy combined controlled vocabulary terms, when available, and free-text terms related to HGS, older adults or aging, and selected aging-related health domains, including cognition, sarcopenia, comorbidity, functional status, and cardiovascular risk. Medical Subject Headings (MeSH) and DeCS terms were used in databases that support controlled vocabulary, while equivalent free-text terms were adapted to the syntax of each database or platform. The strategy was designed to retrieve studies examining HGS across several health-related domains rather than a single disease category. The search terms and combinations used are detailed in Supplementary Appendix 1.

### Selection of studies

The selection process was conducted in multiple stages using Rayyan and Microsoft Excel. Initially, Rayyan facilitated duplicate removal and screening of titles and abstracts based on predefined eligibility criteria. Full-text screening was then performed using a structured Excel matrix that captured key decision fields aligned with the inclusion and exclusion criteria. A pilot calibration phase was implemented to ensure consistency among reviewers: three researchers (DEU, SER, and ZMV) independently screened a random sample of records, and the calibration process concluded once an inter-reviewer agreement of ≥80% was achieved.

Studies were included when eligibility criteria were met and there was agreement among reviewers. In case of discrepancies, a fourth reviewer (DPG), blinded to the previous responses, made the final decision. Eligibility criteria were applied during the full-text analysis for the final selection. Any disagreements regarding eligibility or methodological interpretation were resolved by consensus with a fifth reviewer (JJB). After the scope of the review was refined from comorbidities to clinical conditions and health-related outcomes, age eligibility and conceptual alignment were reassessed according to the revised inclusion criteria.

### Data charting process

Data were extracted using a structured Excel form developed by the authors (DEU, SER, and ZMV). The extraction matrix included first author, year of publication, country, study design, sample size, population setting, age criteria, sex distribution, study objectives, clinical condition or health-related outcome assessed, HGS instrument, HGS measurement protocol, hand assessed when reported, HGS operationalization or cutoff values, and main findings regarding the relationship between HGS and the outcome of interest.

HGS-related variables included the type of dynamometer, hand assessed, posture, number of trials, use of dominant hand or both hands, best value, mean value, combined value, sex-specific thresholds, quartiles, tertiles, quintiles, or continuous analytical units such as per 5-kg change, when available. Extracted outcomes were categorized into health-related domains, including neurocognitive and mental health outcomes, musculoskeletal conditions or syndromes, cardiometabolic or cardiovascular outcomes, functional outcomes, quality-of-life outcomes, frailty or comorbidity-burden outcomes, and adverse health outcomes. Extracted data were reviewed for consistency during team meetings, and discrepancies were resolved by consensus among the reviewers.

### Synthesis of results

Findings were synthesized descriptively and organized according to study characteristics, population setting, study design, health-related domain, HGS measurement and operationalization, and main direction of reported associations. Because of heterogeneity in study designs, populations, HGS protocols, outcome definitions, and analytical approaches, no quantitative synthesis or meta-analysis was performed. Studies were interpreted according to their design, with caution applied to cross-sectional findings, mixed-age samples, and outcomes in which HGS formed part of the diagnostic or screening framework.

### Methodological appraisal

A methodological appraisal of the included studies was conducted to describe the quality and reporting characteristics of the evidence base. Cohort, longitudinal, and panel studies were assessed using the Joanna Briggs Institute Critical Appraisal Checklist for Cohort Studies, whereas cross-sectional studies were assessed using the Appraisal Tool for Cross-Sectional Studies (AXIS). Appraisal results were not used as exclusion criteria but were considered in the interpretation of methodological strengths and limitations across the included literature.

## Results

**FIGURE 1:**
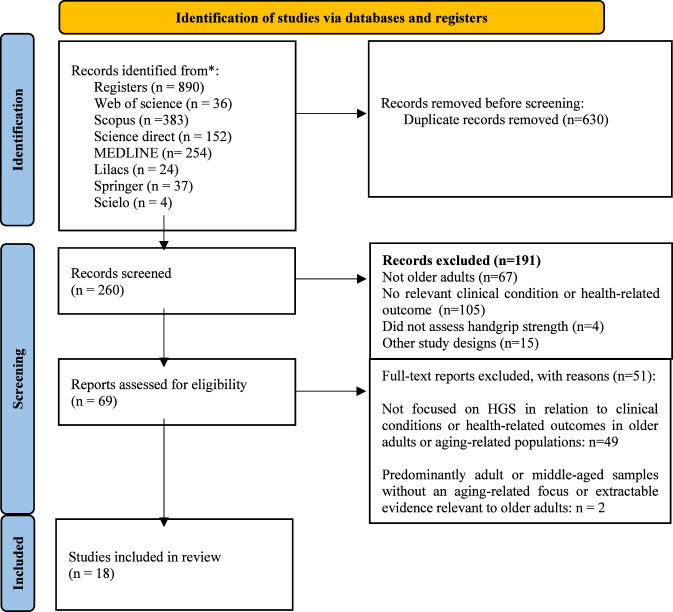
PRISMA-ScR flow diagram.

The study selection process is summarized in the PRISMA-ScR flow diagram (Figure 1). The database search identified 890 records. After duplicate removal and title/abstract screening, 69 full-text reports were assessed for eligibility. Of these, 51 were excluded for not meeting the revised eligibility criteria, and 18 studies were included in the final synthesis.

### Characteristics of publications and population

The 18 studies included in the final synthesis comprised 243,334 participants. Ten studies were cross-sectional (55.6%) (Brooks et al., 2018; Capanema et al., 2022; Chen et al., 2022; Jovanovic et al., 2022; Lin et al., 2021; Moreira et al., 2019; Park et al., 2023; Reeve et al., 2018; Soares et al., 2023; Su et al., 2021), whereas eight used longitudinal, cohort, or panel designs (44.4%) (Chai et al., 2024; Chou et al., 2019; Cui et al., 2024; Duchowny et al., 2022; Kim et al., 2019; McGrath et al., 2020; Peng et al., 2024; Ventura et al., 2023).

Most studies were published between 2019 and 2024 (n = 16; 88.9%) (Capanema et al., 2022; Chai et al., 2024; Chen et al., 2022; Chou et al., 2019; Cui et al., 2024; Duchowny et al., 2022; Jovanovic et al., 2022; Kim et al., 2019; Lin et al., 2021; McGrath et al., 2020; Moreira et al., 2019; Park et al., 2023; Peng et al., 2024; Soares et al., 2023; Su et al., 2021; Ventura et al., 2023).

Table 1 summarizes the main characteristics of the included studies, including publication year, country, sample size, population setting, study design, age, sex distribution, and HGS instrument. Sample sizes ranged from 78 to 190,406 participants. Most studies were conducted in community-dwelling or population-based samples (n = 15; 83.3%) (Brooks et al., 2018; Capanema et al., 2022; Chai et al., 2024; Chen et al., 2022; Chou et al., 2019; Cui et al., 2024; Duchowny et al., 2022; Kim et al., 2019; McGrath et al., 2020; Moreira et al., 2019; Park et al., 2023; Peng et al., 2024; Soares et al., 2023; Ventura et al., 2023), whereas three studies included clinical outpatient, hospital-based, or mixed memory clinic/community samples (16.7%) (Lin et al., 2021; Reeve et al., 2018; Su et al., 2021). The included populations varied substantially in age criteria, ranging from mixed middle-aged-to-older cohorts to studies focused exclusively on adults aged 65 years or older.

**TABLE 1:** Characteristics of the studies.

Author	Year/Country	Sample size, n	Population setting	Study design	Age, years	Sex distribution	HGS instrument
Reeve et al. (2018)	2018/United States	311	Clinical outpatient population	Cross-sectional study	Mean age 69.1 ± 9.5 years	Female 54% Male 46%	Calibrated hydraulic manual dynamometer (Jamar Hand Dynamometer)
Brooks et al. (2018)	2018/United States	3421	Community-dwelling	Cross-sectional study	≥60 years; mean age 69.9 ± 6.9 years	Men 1,660 Women 1761	Dynamometer, model not reported
Chou et al. (2019)	2019/Japan	1,096	Community-dwelling	Prospective cohort study	≥60 years; mean age 69.4 ± 5.8 years	Female 49.1% Male 50.9%	Dynamometer (Takei)
Kim et al. (2019)	2019/Korea	2,378	Community-dwelling	Prospective cohort study	≥65 years; mean age 71.3 ± 5.0 years in men and 71.0 ± 5.1 years in women	Men 1,240 Women 1,138	Hand grip dynamometer (TANITA Hand Grip Meter Blue 6,103)
Moreira et al. (2019)	2019/Brazil	745	Community-dwelling	Cross-sectional study	Mean age 76.6 ± 6.9 years	Women 70.3% Men 29.7%	Dynamometer (JAMAR-J00105)
McGrath et al. (2020)	2020/United States	14,775	Population-based aging cohort	Longitudinal panel study	Mean age 64.1 ± 9.5 years	Women 8,554 Men 6,221	Hand grip dynamometer (spring type, Smedley Scandidact)
Su et al. (2021)	2021/China	1,431	Memory clinic and community-dwelling	Cross-sectional study	Mean age 69.8 ± 9.9 years	Men 48.8% Women 51.2%	Dynamometer (WCS-100)
Lin et al. (2021)	2021/Taiwan	1,007	Hospital-based	Cross-sectional study	Postmenopausal women and men ≥50 years	Women 845 Men 162	Manual digital dynamometer (EH101; Camry)
Duchowny et al. (2022)	2022/United States	190,406	Population-based cohort	Cohort study	39–73 years; mean age 56.5 ± 8.1 years	Women 54% Men 46%	Hand dynamometer (Jamar hydraulic 100,105)
Chen et al. (2022)	2022/Singapore	330	Community-dwelling	Cross-sectional study	Mean age 71.4 ± 8.4 years	Women 182 Men 148	Digital dynamometer (Jamar Plus +)
Jovanovic et al. (2022)	2022/Serbia	98	Community-dwelling	Cross-sectional study	Age between 65 and 85 years	Men 16 Women 82	Standardized dynamometer with tensiometric probe (All4gym d.o.o., Serbia)
Capanema et al. (2022)	2022/Brazil	78	Community-dwelling	Cross-sectional study	Mean age 101.7 ± 2.52 years	Women 55 (70.5%); men 23 (29.5%)	Dynamometer (SAEHAN® model SH5001)
Soares et al. (2023)	2023/Brazil	154	Community-dwelling	Cross-sectional study	65–96 years	Women 100%	Dynamometer (Jamar)
Ventura et al. (2023)	2023/Mexico	2,155	Community-dwelling	Prospective cohort study	≥65 years; mean age 72.4 ± 5.7 years	Women 58% Men 42%	Jamar hydraulic dynamometer, model #5030J1
Park et al. (2023)	2023/Korea	13,966	Population-based survey	Cross-sectional study	>40 years	Men 6,199 Women 7767	Digital grip strength dynamometer (TKK 5401)
Peng et al. (2024)	2024/China	392	Community-dwelling	Prospective cohort study	Mean age 75.77 ± 4.85 years; range 68–90 years	Women 54.3% Men 45.7%	Manual hydraulic dynamometer (Lafayette Instruments)
Chai et al. (2024)	2024/China	4,416	Population-based aging cohort	Longitudinal study	Mean age 67.86 ± 6.57 years	Women 56.7%; men 43.3%	Hand grip dynamometer (hydraulic)
Cui et al. (2024)	2024/China	6,175	Population-based aging cohort	Longitudinal panel study	≥50 years; mean age 69.53 ± 7.57 years	Women 56.44% Men 43.56%	Hand dynamometer (Smedley)

SD (Standard Deviation).

### Health-related domains and HGS measurement

The included studies examined a heterogeneous set of clinical conditions and health-related outcomes grouped into health-related domains. Neurocognitive and mental health outcomes were the most frequently examined domain (n = 13), including depression, cognitive function, cognitive impairment, dementia, Alzheimer’s disease, and cognitive decline (Brooks et al., 2018; Capanema et al., 2022; Chai et al., 2024; Chen et al., 2022; Chou et al., 2019; Cui et al., 2024; Duchowny et al., 2022; Jovanovic et al., 2022; Kim et al., 2019; McGrath et al., 2020; Peng et al., 2024; Su et al., 2021; Ventura et al., 2023). Musculoskeletal conditions or syndromes were assessed in five studies and included sarcopenia, osteoporosis, and psoas-area–based muscle status (Lin et al., 2021; Moreira et al., 2019; Park et al., 2023; Reeve et al., 2018; Soares et al., 2023). Functional outcomes, quality of life, frailty, comorbidity burden, or cardiovascular risk were also addressed across several studies, either as primary outcomes or as part of broader clinical profiles (Capanema et al., 2022; Chai et al., 2024; Chen et al., 2022; Park et al., 2023; Reeve et al., 2018).

HGS measurement varied across studies in terms of dynamometer type, hand assessed, number of attempts, summary value, and operational definition. Six studies used Jamar or Jamar-type dynamometers (n = 6; 33.3%) (Chen et al., 2022; Duchowny et al., 2022; Moreira et al., 2019; Reeve et al., 2018; Soares et al., 2023; Ventura et al., 2023), whereas ten used other specified devices, including Takei, Tanita, Smedley, Camry, Lafayette, Saehan, TKK, WCS-100, or a tensiometric-probe dynamometer (n = 10; 55.6%) (Capanema et al., 2022; Chou et al., 2019; Cui et al., 2024; Jovanovic et al., 2022; Kim et al., 2019; Lin et al., 2021; McGrath et al., 2020; Park et al., 2023; Peng et al., 2024; Su et al., 2021). Two studies did not report a specific dynamometer brand or model (n = 2; 11.1%) (Brooks et al., 2018; Chai et al., 2024).

The operationalization of HGS also differed across studies. Some studies used predefined weakness thresholds, such as <26 kg for men and <16 kg for women (McGrath et al., 2020), <28 kg for men and <18 kg for women (Chen et al., 2022), or <30 kgf for men and <20 kgf for women (Moreira et al., 2019). Other studies used sex-specific quartiles, tertiles, or quintiles, ROC-derived cut-off points, combined HGS, or continuous analytical units such as per 5-kg difference in HGS (Brooks et al., 2018; Chou et al., 2019; Duchowny et al., 2022; Lin et al., 2021; Peng et al., 2024; Su et al., 2021; Ventura et al., 2023). These differences in HGS measurement and operationalization may limit direct comparability across studies and were considered when interpreting the findings.

Across the included studies, lower HGS or HGS-related parameters were reported in relation to poorer neurocognitive, musculoskeletal, functional, and quality-of-life outcomes. Longitudinal studies mainly addressed cognitive decline, cognitive impairment, or dementia-related outcomes, whereas cross-sectional studies more frequently examined depression, sarcopenia, osteoporosis, functional mobility, frailty, or health-related quality of life. Detailed findings by health-related domain and HGS operationalization are presented in Table 2.

**TABLE 2:** Health-related domains, HGS operationalization, and main findings of included studies.

Author	Health-related domain	Specific condition/Outcome	HGS measurement/Operationalization	Main finding related to HGS and health outcome	Methodological or interpretation caution
Reeve et al. (2018)	Cardiovascular/comorbidity burden and frailty	Frailty, comorbidity burden, cardiac risk, and psoas-area–based sarcopenia	Dominant-hand grip strength; frailty defined using age-, sex-, and BMI-adjusted 20th percentile reference values. Frail group HGS: 19.7 ± 6.5 kg	HGS-based frailty was associated with higher Charlson Comorbidity Index, higher Revised Cardiac Risk Index, and lower psoas area	Clinical outpatient vascular population; findings may not generalize to community-dwelling older adults
Brooks et al. (2018)	Neurocognitive and mental health	Depression	Combined HGS calculated as the sum of the highest value from each hand; three trials per hand in standing position. Mean combined HGS: 73.5 kg in men and 46.6 kg in women	Participants with depressive symptoms had significantly lower combined HGS than those without depressive symptoms	Cross-sectional design; directionality between depressive symptoms and lower HGS cannot be inferred
Chou et al. (2019)	Neurocognitive function	10-year cognitive decline	Baseline HGS classified into sex-specific quintiles. Men: Q1 <30.70 kg, Q5 >41.25 kg. Women: Q1 <18.50 kg, Q5 >25.70 kg	Lower HGS was associated with greater decline in MMSE and DSST scores over 10 years	Longitudinal design supports temporality, but residual confounding remains possible
Kim et al. (2019)	Neurocognitive function	Cognitive function/K-MMSE over 8 years	Maximum HGS analyzed longitudinally. Mean maximum HGS: 30.7 kg in men and 19.2 kg in women	Higher baseline HGS was positively associated with baseline and follow-up K-MMSE scores	Cognitive and strength decline may reflect shared aging-related mechanisms
Moreira et al. (2019)	Musculoskeletal conditions/syndromes	Sarcopenia	HGS included as part of sarcopenia classification. Low HGS cutoffs: <30 kgf for men and <20 kgf for women	Sarcopenia prevalence varied according to the HGS, gait speed, and muscle mass cutoff values used	HGS is part of sarcopenia definition; findings may partly reflect criterion overlap
McGrath et al. (2020)	Neurocognitive function	Cognitive impairment and HGS decline	HGS measured longitudinally. Weak HGS defined as <26 kg for men and <16 kg for women; associations also analyzed per 5-kg difference	Higher HGS was associated with lower odds of future cognitive impairment; cognitive impairment also predicted subsequent HGS decline	Bidirectional association; causal direction cannot be assumed
Su et al. (2021)	Neurocognitive function	Mild cognitive impairment, Alzheimer’s disease, and cognitive function	HGS cutoffs estimated by sex and age group. For MCI: women >70 years 17.5 kg and ≤70 years 21.9 kg; men >70 years 25.8 kg and ≤70 years 36.2 kg	Stronger HGS was associated with better cognitive performance; HGS was evaluated as a possible screening measure for MCI and AD.	Clinical/community cognitive sample; proposed screening cutoffs require validation
Lin et al. (2021)	Musculoskeletal conditions	Osteoporosis	ROC-derived thresholds for osteoporosis discrimination: 21.9 kg in women and 28.7 kg in men	HGS was related to bone mineral density and bone microarchitecture and was associated with osteoporosis status/risk in this cross-sectional sample	Cross-sectional hospital-based sample; “prediction” should be interpreted as diagnostic discrimination, not longitudinal prediction
Duchowny et al. (2022)	Neurocognitive function and adverse health outcomes	Dementia, cognition, and neuroimaging outcomes	HGS assessed in both hands; maximum value from right/left hand used; estimates reported per 5-kg decrement in HGS	Lower HGS was associated with incident dementia, poorer cognition, and worse neuroimaging markers	Mixed middle-aged/older cohort; not exclusively older adults
Chen et al. (2022)	Neurocognitive and functional outcomes	Cognitive function and functional mobility	Low HGS defined as <28 kg for men and <18 kg for women; HGS asymmetry defined as >10% stronger grip in either hand	Low HGS was associated with poorer cognition and slower functional mobility; asymmetry alone showed less consistent associations	Cross-sectional design; associations may reflect shared functional decline
Jovanovic et al. (2022)	Neurocognitive and mental health	Cognitive abilities and depressive symptoms	Dominant and non-dominant hands assessed; variables included maximal force, maximal rate of force development, and endurance at 50% maximal force	Better cognitive performance was associated with better HGS-related parameters	Small sample and marked sex imbalance; limited generalizability
Capanema et al. (2022)	Functional and neurocognitive outcomes	Functional ability and cognitive status in centenarians	Right and left HGS assessed with manual dynamometry. Mean HGS: right hand 12.6 ± 6.2 kg; left hand 10.73 ± 6.3 kg	Centenarians with preserved cognition and better functional capacity had higher right and left HGS.	Cross-sectional centenarian sample; findings may not generalize to younger older adults
Soares et al. (2023)	Musculoskeletal conditions/syndromes	Sarcopenia	HGS evaluated as a discriminator for sarcopenia; ROC-derived cutoff ≤20 kg for older Brazilian women	HGS showed good discrimination for identifying sarcopenia	HGS is part of sarcopenia screening/diagnosis; external validation of cutoffs is needed
Ventura et al. (2023)	Neurocognitive function	Cognitive impairment over 20 years	Dominant-hand HGS; two attempts; highest value used. Sex-specific quartiles: men Q1 <22 kg and Q4 ≥35 kg; women Q1 <14 kg and Q4 ≥22.5 kg	Higher HGS quartiles were associated with lower odds of cognitive impairment over time	Findings are specific to older Mexican Americans; attrition and mortality may influence estimates
Park et al. (2023)	Musculoskeletal and quality-of-life outcomes	Osteoporosis and health-related quality of life	HGS measured with a digital grip strength dynamometer (TKK 5401) and categorized into weak and strong HGS groups. Mean HGS: 21 kg in the weak-HGS group and 34 kg in the strong-HGS group	Participants with osteoporosis and weak HGS had poorer HRQoL than those without osteoporosis and with strong HGS.	Cross-sectional >40-year sample; not exclusively older adults
Peng et al. (2024)	Neurocognitive function	Cognitive impairment and HGS asymmetry	Low HGS and HGS asymmetry assessed. Weakest grip strength tertile: men 13.0–27.5 kg; women 4.0–16.0 kg	Coexisting low HGS and HGS asymmetry was associated with cognitive impairment over time, especially in men	Sex-specific findings; relatively small cohort
Chai et al. (2024)	Neurocognitive and functional outcomes	Cognitive function and functional limitation	HGS analyzed longitudinally. Mean HGS: 27.69 ± 8.94 kg. Functional limitation tested as mediator	HGS was positively associated with subsequent cognitive function and indirectly related through functional limitation	Mediation was based on observational data; causal pathway should be interpreted cautiously
Cui et al. (2024)	Neurocognitive function	Bidirectional longitudinal relationship between grip strength and cognition	Grip strength and multiple cognitive domains analyzed using cross-lagged panel models. Baseline grip strength: men 43.00 kg [37.00–49.00], women 26.00 kg [22.00–30.00]	Grip strength and cognitive function showed statistically significant bidirectional longitudinal associations	Mixed ≥50-year aging cohort; bidirectionality suggests reciprocal associations, but mechanisms cannot be inferred

HGS, handgrip strength; BMI, body mass index; CCI, charlson comorbidity index; RCRI, revised cardiac risk index; MMSE, Mini-Mental State Examination; DSST, digit symbol substitution test; K-MMSE, Korean Mini-Mental State Examination; MCI, mild cognitive impairment; AD, Alzheimer’s disease; BMD, bone mineral density; HRQoL, health-related quality of life; ROC, receiver operating characteristic.

Sarcopenia-related findings should be interpreted with caution because HGS, is part of several sarcopenia screening or diagnostic frameworks.

### Methodological appraisal

The methodological appraisal of the included studies is presented in Supplementary Appendix 2. The eight longitudinal, cohort, or panel studies were assessed using the JBI Critical Appraisal Checklist for Cohort Studies (Supplementary Table A1) (Chai et al., 2024; Chou et al., 2019; Cui et al., 2024; Duchowny et al., 2022; Kim et al., 2019; McGrath et al., 2020; Peng et al., 2024; Ventura et al., 2023). The ten cross-sectional studies were assessed using the AXIS tool (Supplementary Table A2).

Overall, most studies adequately reported their objectives, target populations, measurement procedures, and statistical analyses. The main recurrent limitations were incomplete reporting of some HGS protocol components, heterogeneity in outcome definitions and HGS operationalization, limited information on non-response or attrition in some studies, and the inherent limitation of cross-sectional designs for interpreting directionality. Among longitudinal studies, prospective or repeated-measures designs strengthened temporal interpretation, although attrition, residual confounding, and differences in follow-up duration remained relevant methodological considerations.

## Discussion

This scoping review mapped the available evidence on handgrip strength (HGS) in relation to clinical conditions and health-related outcomes in older adults and aging-related populations. The included studies showed that HGS has been examined mainly in relation to neurocognitive and mental health outcomes, followed by musculoskeletal conditions, functional outcomes, quality of life, frailty, comorbidity burden, and cardiovascular risk. Overall, the literature indicates that HGS has been used as a simple and widely available functional measure to characterize different health domains in later life (Bohannon, 2015; Roberts et al., 2011; Tomkinson et al., 2025). However, the predominance of observational designs, population heterogeneity, variability in HGS measurement protocols, and differences in outcome definitions limit direct comparability across studies and preclude causal interpretation.

The neurocognitive domain was the most frequently examined. Several longitudinal studies reported associations between lower baseline HGS and subsequent decline in cognitive function, cognitive impairment, or dementia-related outcomes (Chai et al., 2024; Chou et al., 2019; Cui et al., 2024; Duchowny et al., 2022; Kim et al., 2019; McGrath et al., 2020; Peng et al., 2024; Ventura et al., 2023). This pattern may be compatible with the hypothesis that HGS reflects broader aspects of functional status in aging; however, the included studies do not allow determination of whether these associations are explained by shared mechanisms, reciprocal relationships, or residual confounding. Some studies also suggest reciprocal associations between cognitive function and muscle strength, indicating that lower HGS may not only precede cognitive decline but may also occur in parallel with, or as a consequence of, other aging-related processes (Clouston et al., 2013; Cui et al., 2024; McGrath et al., 2020). Therefore, it is more appropriate in this field to refer to longitudinal or reciprocal associations rather than direct causal prediction. Future studies should incorporate repeated HGS assessments and domain-specific cognitive measures to better clarify the temporality and direction of these associations.

Musculoskeletal outcomes were also frequently addressed, particularly sarcopenia and osteoporosis. Sarcopenia-related findings require particular caution because HGS is included in several screening algorithms and diagnostic frameworks for sarcopenia (Chen et al., 2020; Cruz-Jentoft et al., 2019). Herefore, associations between HGS and sarcopenia may partly reflect overlap between the exposure and the outcome rather than an independent relationship between HGS and a separate clinical condition. In this regard, HGS may be useful as a functional component of sarcopenia assessment, but it should not be presented in this review as an independent marker of sarcopenia without acknowledging this conceptual overlap. For osteoporosis, the included studies reported associations between HGS and bone-related measures or health-related quality of life; however, these findings were mainly derived from cross-sectional studies or mixed-age samples, limiting temporal interpretation (Lin et al., 2021; Park et al., 2023).

One of the most relevant methodological findings of this review was the variability in HGS measurement and operationalization. The included studies used different types of dynamometers, including Jamar or Jamar-type devices, Takei, Smedley, Camry, Tanita, Lafayette, Saehan, TKK, WCS-100, and tensiometric-probe devices. They also differed in the hand assessed, number of attempts, body position, use of maximum, mean, or combined values, and categorization of strength using cut-off points, tertiles, quartiles, quintiles, or continuous units such as per 5-kg change (Roberts et al., 2011; Sousa-Santos and Amaral, 2017). This heterogeneity limits direct comparability across studies and may affect the magnitude, precision, and interpretation of the reported associations. Recent international normative evidence has also emphasized the importance of standardized HGS measurement and interpretation according to age, sex, and body characteristics, particularly when HGS is used for screening, population surveillance, or between-group comparisons (Tomkinson et al., 2025).

Variation in HGS cut-off points also deserves attention. Some studies used previously established thresholds to define low HGS, whereas others proposed ROC-derived values, age- and sex-specific cut-off points, or relative categories within the study sample. This diversity may reflect true population differences, but it also limits the direct transferability of thresholds across countries, age groups, and clinical settings (Lera et al., 2018; Ramírez-Vélez et al., 2019; Tomkinson et al., 2025). Differences by sex and age should therefore not be discussed as isolated findings, but rather as methodological elements that are necessary for appropriate interpretation of HGS. Future studies should report results stratified by sex and age, describe the measurement protocol in detail, and justify the choice of cut-off points. Studies validating context-specific thresholds in underrepresented populations and specific clinical settings are also needed.

Methodological appraisal also provides an important element for interpreting the findings. The use of design-specific appraisal tools, such as the JBI checklist for cohort and longitudinal studies and the AXIS tool for cross-sectional studies, allowed recurrent limitations related to follow-up, non-response, confounding control, and the completeness of methodological reporting to be identified. The JBI checklist assesses key aspects of cohort studies, including exposure measurement, identification and management of confounding factors, completeness of follow-up, and appropriateness of statistical análisis (Barker et al., 2025). In turn, AXIS was developed through a Delphi consensus process to assess cross-sectional studies in areas such as clarity of objectives, sample representativeness, measurement of variables, non-response, statistical analysis, limitations, conflicts of interest, and ethical approval (Downes et al., 2016). In this review, these appraisal results were not used as exclusion criteria, but rather to support interpretation of the methodological robustness and limitations of the available evidence.

The characteristics of the included populations also affect the applicability of the findings. Most studies were conducted in community-dwelling populations or population-based cohorts, although some included clinical, hospital-based, or memory-clinic samples. In addition, several studies included mixed samples of middle-aged and older adults; therefore, their findings should not be extrapolated to older adults exclusively without caution. This population heterogeneity was one of the reasons for distinguishing studies focused on older adults from studies of aging-related populations. Within the studies included in this review, Latin American representation was limited, with studies from Brazil and Mexican-American populations, but no studies conducted in Colombia. This does not imply an absence of regional research, since population-specific reference values have been reported in Colombian and Chilean older adults (Lera et al., 2018; Ramírez-Vélez et al., 2019). Rather, it indicates that Latin American evidence was less represented among the studies meeting the eligibility criteria of this review. Further regional studies on HGS and health-related outcomes in older adults are warranted, particularly considering sociodemographic, functional, clinical, and healthcare-access differences.

This review has several strengths. First, it adopted a broader and more conceptually appropriate classification of outcomes, organized around clinical conditions and health-related outcomes rather than a narrow comorbidity framework. Second, it included studies published in English, Spanish, and Portuguese, which broadened the scope of the search beyond English-language literature. Third, eligibility was reassessed to ensure coherence among the target population, the revised scope, and the included studies. Fourth, data extraction included not only health-related outcomes, but also HGS instruments, measurement protocols, and operational definitions. Finally, methodological appraisal using JBI and AXIS helped identify recurrent strengths and limitations across longitudinal and cross-sectional designs.

Several limitations should also be acknowledged. Heterogeneity in study designs, populations, instruments, measurement protocols, cut-off points, and outcome definitions precluded quantitative synthesis. Most included studies were observational; therefore, the reported associations cannot be interpreted causally. Some studies included mixed samples of middle-aged and older adults, which may affect the specificity of the findings for older populations. In addition, as expected in a scoping review, the main purpose was to map the extent, range, and nature of the evidence rather than to estimate pooled effects or formulate definitive clinical recommendations. These limitations highlight the need for longitudinal studies using standardized HGS protocols, consistent outcome definitions, and population-specific validation of cut-off points.

## Conclusion

This scoping review mapped evidence on HGS across clinical conditions and health-related outcomes in older adults and aging-related populations. Most studies focused on neurocognitive outcomes, followed by musculoskeletal, functional, quality-of-life, frailty-related, cardiovascular, and comorbidity-burden outcomes. Lower HGS was commonly reported in relation to poorer health-related outcomes, but heterogeneity in study design, population characteristics, HGS protocols, and outcome definitions limits direct comparability and causal interpretation. Further longitudinal studies using standardized HGS measurement protocols and consistent outcome definitions are needed.
