# Supplementary material for: Handgrip strength across clinical conditions and health-related outcomes in older adults: a scoping review
Source: Front Aging. 2026 Jun 30;7:1804033. doi: 10.3389/fragi.2026.1804033 (PMC13364915; doi:10.3389/fragi.2026.1804033)
Supplement: Supplementary file 1 [file Supplementaryfile1.docx]

**PRISMA-ScR Checklist**

| **Section** | **Item** | **PRISMA-ScR checklist item** | **Reported location / comments** |
| --- | --- | --- | --- |
| Title | 1 | Identify the report as a scoping review. | Reported in the title: “Handgrip Strength Across Clinical Conditions and Health-Related Outcomes in Older Adults: A Scoping Review.” |
| Abstract — Structured summary | 2 | Provide a structured summary that includes, as applicable, background, objectives, eligibility criteria, sources of evidence, charting methods, results, and conclusions that relate to the review questions and objectives. | Reported in the Abstract under Background and Aim, Methods, Results, and Conclusion. |
| Introduction — Rationale | 3 | Describe the rationale for the review in the context of what is already known. Explain why the review questions/objectives lend themselves to a scoping review approach. | Reported in the Introduction, where the dispersed literature on HGS across clinical conditions and health-related outcomes is described, together with the need for an evidence-mapping approach. |
| Introduction — Objectives | 4 | Provide an explicit statement of the questions and objectives being addressed with reference to their key elements. | Reported at the end of the Introduction and in the Methods under Study design, including the review question. |
| Methods — Protocol and registration | 5 | Indicate whether a review protocol exists; state if and where it can be accessed; and, if available, provide registration information. | Reported in Methods, Study design. The review protocol was not registered in a publicly accessible registry. |
| Methods — Eligibility criteria | 6 | Specify characteristics of the sources of evidence used as eligibility criteria and provide a rationale. | Reported in Methods, Eligibility criteria, using the PCC framework. Eligibility criteria included population, concept, context, study designs, language, and publication type. |
| Methods — Information sources | 7 | Describe all information sources in the search, including databases, dates of coverage, and the date the most recent search was executed. | Reported in Methods, Sources of information and search strategy, and Appendix 1. Searches were conducted in March 2024 in ScienceDirect, Scopus, Web of Science, LILACS, SciELO, MEDLINE via PubMed, and SpringerLink as a supplementary publisher platform. |
| Methods — Search | 8 | Present the full electronic search strategy for at least one database, including any limits used, such that it could be repeated. | Reported in Appendix 1 as search terms, search combinations, database/platforms, dates, and records retrieved. The combinations were adapted according to the syntax and controlled vocabulary options of each database/platform. |
| Methods — Selection of sources of evidence | 9 | State the process for selecting sources of evidence, including screening and eligibility, included in the scoping review. | Reported in Methods, Selection of Studies. Rayyan and Microsoft Excel were used, with pilot calibration, independent screening, full-text assessment, and resolution of discrepancies by reviewers. |
| Methods — Data charting process | 10 | Describe the methods of charting data from the included sources of evidence and any processes for obtaining and confirming data from investigators. | Reported in Methods, Data charting process. A structured Excel form was used, and extracted data were reviewed for consistency during team meetings. |
| Methods — Data items | 11 | List and define all variables for which data were sought and any assumptions and simplifications made. | Reported in Methods, Data charting process. Variables included author, year, country, study design, sample size, population setting, age, sex, clinical condition or health-related outcome, HGS instrument, protocol, hand assessed, operationalization/cut-off values, and main findings. |
| Methods — Critical appraisal of individual sources of evidence | 12 | If done, provide a rationale for conducting a critical appraisal of included sources of evidence; describe the methods used and how this information was used in any data synthesis. | Reported in Methods, Methodological appraisal; Results, Methodological appraisal; and Appendix 2. JBI was used for cohort, longitudinal, and panel studies, and AXIS for cross-sectional studies. Appraisal was not used as an exclusion criterion. |
| Methods — Summary measures | 13 | Not applicable for scoping reviews. | Not applicable. No meta-analysis or pooled effect estimate was conducted. |
| Methods — Synthesis of results | 14 | Describe the methods of handling and summarizing the data that were charted. | Reported in Methods, Synthesis of results. Findings were synthesized descriptively by study characteristics, population setting, health-related domain, HGS measurement/operationalization, and direction of associations. |
| Methods — Risk of bias across studies | 15 | Not applicable for scoping reviews. | Not applicable. Risk of bias across studies was not assessed. Methodological appraisal was used descriptively. |
| Methods — Additional analyses | 16 | Not applicable for scoping reviews. | Not applicable. No additional quantitative analyses were performed. |
| Results — Selection of sources of evidence | 17 | Give numbers of sources screened, assessed for eligibility, and included in the review, with reasons for exclusions at each stage, ideally using a flow diagram. | Reported in Results and Figure 1, PRISMA-ScR flow diagram. The search identified 890 records; 69 full-text reports were assessed; 18 studies were included. |
| Results — Characteristics of sources of evidence | 18 | For each source of evidence, present characteristics for which data were charted and provide the citations. | Reported in Results and Table 1. Characteristics include author, year/country, sample size, population setting, study design, age, sex distribution, and HGS instrument. |
| Results — Critical appraisal within sources of evidence | 19 | If done, present data on critical appraisal of included sources of evidence. | Reported in Results, Methodological appraisal, and Appendix 2. JBI and AXIS appraisal results are presented in Tables 3 and 4. |
| Results — Results of individual sources of evidence | 20 | For each included source of evidence, present the relevant data that were charted and that relate to the review questions and objectives. | Reported in Table 2, including health-related domain, specific condition/outcome, HGS measurement/operationalization, main finding related to HGS and health outcome, and methodological or interpretation caution. |
| Results — Synthesis of results | 21 | Summarize and/or present the charting results as they relate to the review questions and objectives. | Reported in Results, especially Health-related domains and HGS measurement, and in Table 2. |
| Results — Risk of bias across studies | 22 | Not applicable for scoping reviews. | Not applicable. |
| Results — Additional analyses | 23 | Not applicable for scoping reviews. | Not applicable. |
| Discussion — Summary of evidence | 24 | Summarize the main results, including an overview of concepts, themes, and types of evidence available, link to the review questions and objectives, and consider relevance to key groups. | Reported in the Discussion. The review summarizes evidence across neurocognitive, musculoskeletal, functional, quality-of-life, frailty-related, cardiovascular, and comorbidity-burden outcomes, with emphasis on methodological heterogeneity and interpretation. |
| Discussion — Limitations | 25 | Discuss the limitations of the scoping review process. | Reported in the Discussion. Limitations include heterogeneity in study designs, populations, instruments, HGS protocols, cut-off points, outcome definitions, and the predominance of observational evidence. |
| Discussion — Conclusions | 26 | Provide a general interpretation of the results with respect to the review questions and objectives, as well as potential implications and/or next steps. | Reported in the Conclusion. The review emphasizes the descriptive and exploratory nature of the evidence and the need for longitudinal studies using standardized HGS protocols and consistent outcome definitions. |
| Funding | 27 | Describe sources of funding for the included sources of evidence, as well as sources of funding for the scoping review. Describe the role of the funders of the scoping review. | Reported in the Title page under Funding. The review was funded by Dirección General de Investigaciones of Universidad Santiago de Cali under call No. DGI-01–2026 and project 442-621123-385. |
