# Supplementary material for: Handgrip strength across clinical conditions and health-related outcomes in older adults: a scoping review
Source: Front Aging. 2026 Jun 30;7:1804033. doi: 10.3389/fragi.2026.1804033 (PMC13364915; doi:10.3389/fragi.2026.1804033)
Supplement: Supplementary file 2 [file Table1.DOCX]

**APPENDIX 1. Search strategies across databases and platforms**

The search strategy was designed to identify studies examining handgrip strength in older adults or aging-related populations across several health-related domains. Searches combined terms related to handgrip strength, aging, and selected clinical or functional outcomes. Controlled vocabulary was used when available, and free-text terms were adapted according to the syntax and search options of each database or platform.

**Summary of searches**

| **Database/platform** | **Search terms used** | **Controlled vocabulary / free-text** | **Search date** | **Records retrieved** |
| --- | --- | --- | --- | --- |
| Scopus | Combinations of terms for elderly/geriatrics, hand strength/handgrip strength, and selected health-related domains | Free-text | March 11, 2024 | 383 |
| Web of Science | Combinations of terms for elderly/geriatrics, hand strength/handgrip strength, and selected health-related domains | Free-text | March 11, 2024 | 36 |
| ScienceDirect | Combinations of terms for elderly/geriatrics, hand strength/handgrip strength, and selected health-related domains | Free-text | March 11, 2024 | 152 |
| MEDLINE via PubMed | Combinations of MeSH and free-text terms for hand strength, aging, and selected health-related domains | MeSH + free-text | March 12, 2024 | 254 |
| SpringerLink* | Combinations of terms for elderly/geriatrics, hand strength/handgrip strength, and selected health-related domains | Free-text | March 12, 2024 | 37 |
| LILACS | Combinations of DeCS and free-text terms for hand strength, aging, and selected health-related domains | DeCS + free-text | March 13, 2024 | 24 |
| SciELO | Combinations of terms for elderly/geriatrics, hand strength/handgrip strength, and selected health-related domains | Free-text | March 13, 2024 | 4 |

**SpringerLink was used as a supplementary publisher platform to identify potentially relevant full-text articles from Springer journals and was not treated as a bibliographic database.*

**Search terms**

The following controlled vocabulary and free-text terms were used according to the options available in each database or platform.

**Controlled vocabulary terms, when available:** Hand Strength, Aged, Sarcopenia, Cognition, Comorbidity.

**Free-text terms:** elderly, geriatrics, handgrip strength, hand strength, grip strength, muscle strength, functional status, physical function, cardiovascular risk.

**Search combinations**

The search was organized around three groups of terms: aging-related terms, handgrip strength or muscle strength terms, and terms related to selected health domains. The following combinations were used and adapted across databases and platforms:

1. Elderly AND Hand Strength AND Sarcopenia
2. Elderly AND Hand Strength AND Comorbidity
3. Elderly AND Hand Strength AND Cognition
4. Geriatrics AND Hand Strength AND Physical function
5. Elderly AND Hand Strength AND Functional status
6. Elderly AND Hand Strength AND Cardiovascular risk
7. Elderly AND Muscle Strength AND Sarcopenia

**Notes**

The search included sarcopenia and comorbidity as part of a broader set of health-related domains, not as the only conceptual framework for the review. During study selection and data charting, eligible studies were classified according to the revised scope of the review: clinical conditions and health-related outcomes in older adults and aging-related populations.

Because each database and platform has different indexing systems and search interfaces, the combinations were adapted to the available controlled vocabulary, free-text fields, and syntax of each source. The total number of records retrieved across all databases and platforms was 890.
