# Supplementary material for: Handgrip strength across clinical conditions and health-related outcomes in older adults: a scoping review
Source: Front Aging. 2026 Jun 30;7:1804033. doi: 10.3389/fragi.2026.1804033 (PMC13364915; doi:10.3389/fragi.2026.1804033)
Supplement: Supplementary file 3 [file Table2.DOCX]

**APPENDIX 2.** Methodological appraisal of included studies

**Table 3. JBI Critical Appraisal Checklist for Cohort Studies**

| **Study** | **JBI 1** | **JBI 2** | **JBI 3** | **JBI 4** | **JBI 5** | **JBI 6** | **JBI 7** | **JBI 8** | **JBI 9** | **JBI 10** | **JBI 11** |
| --- | --- | --- | --- | --- | --- | --- | --- | --- | --- | --- | --- |
| Chou et al. | Yes | Yes | Yes | Yes | Yes | Yes | Yes | Yes | Unclear | Yes | Yes |
| Kim et al. | Yes | Yes | Yes | Yes | Yes | Yes | Yes | Yes | Unclear | Yes | Yes |
| McGrath et al. | Yes | Yes | Yes | Yes | Yes | Yes | Yes | Yes | Unclear | Yes | Yes |
| Duchowny et al. | Yes | Yes | Yes | Yes | Yes | Yes | Yes | Yes | Yes | Yes | Yes |
| Ventura et al. | Yes | Yes | Yes | Yes | Yes | Yes | Yes | Yes | Unclear | Yes | Yes |
| Peng et al. | Yes | Yes | Yes | Yes | Yes | Yes | Yes | Yes | Unclear | Yes | Yes |
| Chai et al. | Yes | Yes | Yes | Yes | Yes | Unclear | Yes | Yes | Unclear | Yes | Yes |
| Cui et al. | Yes | Yes | Yes | Yes | Yes | Unclear | Yes | Yes | No | No | Yes |

*JBI: Joanna Briggs Institute. Yes: criterion met; Unclear: insufficient information reported or not fully described; No: criterion not met. Cohort, longitudinal, and panel studies were appraised using the JBI Critical Appraisal Checklist for Cohort Studies. The appraisal was used to describe methodological quality and was not applied as an exclusion criterion.*

**Checklist items**

JBI 1. Were the two groups similar and recruited from the same population?

JBI 2. Were the exposures measured similarly to assign people to both exposed and unexposed groups?

JBI 3. Was the exposure measured in a valid and reliable way?

JBI 4. Were confounding factors identified?

JBI 5. Were strategies to deal with confounding factors stated?

JBI 6. Were the groups/participants free of the outcome at the start of the study or at the moment of exposure?

JBI 7. Were the outcomes measured in a valid and reliable way?

JBI 8. Was the follow-up time reported and sufficient to be long enough for outcomes to occur?

JBI 9. Was follow-up complete, and if not, were the reasons for loss to follow-up described and explored?

JBI 10. Were strategies to address incomplete follow-up utilized?

JBI 11. Was appropriate statistical analysis used?

**Table 4.** Appraisal Tool for Cross-Sectional Studies (AXIS)

| **Study** | **AXIS 1** | **AXIS 2** | **AXIS 3** | **AXIS 4** | **AXIS 5** | **AXIS 6** | **AXIS 7** | **AXIS 8** | **AXIS 9** | **AXIS 10** | **AXIS 11** | **AXIS 12** | **AXIS 13** | **AXIS 14** | **AXIS 15** | **AXIS 16** | **AXIS 17** | **AXIS 18** | **AXIS 19** | **AXIS 20** |
| --- | --- | --- | --- | --- | --- | --- | --- | --- | --- | --- | --- | --- | --- | --- | --- | --- | --- | --- | --- | --- |
| Reeve et al. | Yes | Yes | Unclear | Yes | Yes | Unclear | Unclear | Yes | Yes | Yes | Yes | Yes | Unclear | No | Yes | Yes | Yes | Yes | Unclear | Yes |
| Brooks et al. | Yes | Yes | Yes | Yes | Yes | Yes | Yes | Yes | Yes | Yes | Yes | Yes | Unclear | Unclear | Yes | Yes | Yes | Yes | Yes | Yes |
| Moreira et al. | Yes | Yes | Unclear | Yes | Yes | Yes | Unclear | Yes | Yes | Yes | Yes | Yes | Unclear | No | Yes | Yes | Yes | Yes | Yes | Yes |
| Su et al. | Yes | Yes | Unclear | Yes | Yes | Unclear | Unclear | Yes | Yes | Yes | Yes | Yes | Unclear | Unclear | Yes | Yes | Yes | Yes | Yes | Yes |
| Lin et al. | Yes | Yes | Unclear | Yes | Yes | Unclear | Unclear | Yes | Yes | Yes | Yes | Yes | Unclear | No | Yes | Yes | Yes | Yes | Unclear | Yes |
| Chen et al. | Yes | Yes | Unclear | Yes | Yes | Yes | Unclear | Yes | Yes | Yes | Yes | Yes | Unclear | Unclear | Yes | Yes | Yes | Yes | Yes | Yes |
| Jovanovic et al. | Yes | Yes | Unclear | Yes | Unclear | Unclear | Unclear | Yes | Yes | Yes | Yes | Yes | Unclear | No | Yes | Yes | Yes | Yes | Yes | Yes |
| Capanema et al. | Yes | Yes | Unclear | Yes | Yes | Unclear | Unclear | Yes | Yes | Yes | Yes | Yes | Unclear | No | Yes | Yes | Yes | Yes | Yes | Yes |
| Soares et al. | Yes | Yes | Unclear | Yes | Yes | Unclear | Unclear | Yes | Yes | Yes | Yes | Yes | Unclear | Unclear | Yes | Yes | Yes | Yes | Yes | Yes |
| Park et al. | Yes | Yes | Yes | Yes | Yes | Yes | Yes | Yes | Yes | Yes | Yes | Yes | Unclear | Unclear | Yes | Yes | Yes | Yes | Unclear | Yes |

*AXIS: Appraisal Tool for Cross-Sectional Studies. Yes: criterion met; Unclear: insufficient information reported or not fully described; No: criterion not met. Cross-sectional studies were appraised using the AXIS tool. The appraisal was used to describe methodological quality and was not applied as an exclusion criterion.*

**Checklist items**

AXIS 1. Were the aims/objectives of the study clear?

AXIS 2. Was the study design appropriate for the stated aim(s)?

AXIS 3. Was the sample size justified?

AXIS 4. Was the target/reference population clearly defined?

AXIS 5. Was the sample frame taken from an appropriate population base so that it closely represented the target/reference population under investigation?

AXIS 6. Was the selection process likely to select subjects/participants that were representative of the target/reference population under investigation?

AXIS 7. Were measures undertaken to address and categorize non-responders?

AXIS 8. Were the risk factor and outcome variables measured appropriate to the aims of the study?

AXIS 9. Were the risk factor and outcome variables measured correctly using instruments/measurements that had been trialed, piloted, or published previously?

AXIS 10. Is it clear what was used to determine statistical significance and/or precision estimates?

AXIS 11. Were the methods, including statistical methods, sufficiently described to enable them to be repeated?

AXIS 12. Were the basic data adequately described?

AXIS 13. Does the response rate raise concerns about non-response bias?

AXIS 14. If appropriate, was information about non-responders described?

AXIS 15. Were the results internally consistent?

AXIS 16. Were the results for the analyses described in the methods presented?

AXIS 17. Were the authors’ discussions and conclusions justified by the results?

AXIS 18. Were the limitations of the study discussed?

AXIS 19. Were there any funding sources or conflicts of interest that may affect the authors’ interpretation of the results?

AXIS 20. Was ethical approval or consent of participants attained
